# Stop Worrying about Multiple-Choice: Fact Knowledge Does Not Change with Response Format

**DOI:** 10.3390/jintelligence10040102

**Published:** 2022-11-14

**Authors:** Benjamin Goecke, Marlena Staab, Catherine Schittenhelm, Oliver Wilhelm

**Affiliations:** Institute for Psychology and Pedagogy, Ulm University, Albert-Einstein-Allee 47, 89081 Ulm, Germany

**Keywords:** crystallized intelligence, response formats, declarative knowledge

## Abstract

Declarative fact knowledge is a key component of crystallized intelligence. It is typically measured with multiple-choice (MC) items. Other response formats, such as open-ended formats are less frequently used, although these formats might be superior for measuring crystallized intelligence. Whereas MC formats presumably only require recognizing the correct response to a question, open-ended formats supposedly require cognitive processes such as searching for, retrieving, and actively deciding on a response from long-term memory. If the methods of inquiry alter the cognitive processes involved, mean-changes between methods for assessing declarative knowledge should come along with changes in the covariance structure. We tested these assumptions in two online studies administering declarative knowledge items in different response formats (MC, open-ended, and open-ended with cues). Item difficulty clearly increases in the open-ended methods although effects in logistic regression models vary slightly across items. Importantly, latent variable analyses suggest that the method of inquiry does not affect what is measured with different response formats. These findings clearly endorse the position that crystallized intelligence does not change as a function of the response format.

## 1. Introduction

Fact knowledge questions are frequently used to assess students’ learning progress and serve as a default procedure for assessing declarative knowledge as a pivotal component of crystallized intelligence (Gc; c.f. Wilhelm and Kyllonen 2021). Prototypically, such questions are administered in multiple-choice (MC) formats. However, alternative open response formats are regularly suggested to overcome possible disadvantages of MC response formats such as fostering recognition in contrast to actual retrieval of information from long-term memory (LTM) (e.g., Becker and Johnston 1999). In fact, open-ended response formats have been argued to measure distinct cognitive processes as compared to what is measured with MC formats (e.g., Becker and Johnston 1999; Hickson and Reed 2011; Krieg and Uyar 2001), but the evidence regarding this supposition is elusive (e.g., Scully 2017) and should be brought into perspective.

With the current studies, we contrast three competing response formats for assessing declarative knowledge as understood by extensions of contemporary intelligence structure models (Carroll 1993; McGrew 2005, 2009; Wilhelm and Kyllonen 2021). Study 1 aimed at contributing to a better understanding of how different response formats affect the empirical difficulties of conventional fact knowledge items and to what extent mean-changes in empirical difficulties between response formats of items otherwise equivalent occur. In addition to that, in Study 2 we explored the consequences of different response formats on the covariance structure of the tests from a multi-trait multi-method perspective of individual differences (Campbell and Fiske 1959). In the next sections, we embed declarative knowledge into the framework of crystallized intelligence, describe different modes of its measurement, discuss their advantages and disadvantages in application, and further describe the possible underlying cognitive processes required by the different response formats.

### 1.1. Declarative Knowledge as an Indicator of Crystallized Intelligence

The study of knowledge complements efforts to understand and explain individual differences in human cognitive abilities (Cattell 1957), and especially crystallized intelligence (Gc). Gc is “typically described as a person’s breadth and depth of acquired knowledge of the language, information and concepts of a specific culture, and/or the application of this knowledge” (McGrew 2009, p. 5). Although a broad Gc factor is proposed in contemporary intelligence structure models (Carroll 1993; McGrew 2005, 2009), it was argued that conventional measurements of Gc fail to include assessments of fact knowledge, although fact knowledge should be understood as a key component of Gc (Wilhelm and Kyllonen 2021). Knowledge regarding broad and general content domains, both curricular and extracurricular (Cattell 1971; Schipolowski et al. 2014; Wilhelm and Kyllonen 2021), should also be deemed the result of ongoing acculturation processes (Ackerman 2000) and of intellectual investment traits (e.g., von Stumm and Ackerman 2013).

Tests of fact knowledge have been shown to load highly on a general factor of Gc, which was marked by verbal and language-related abilities (e.g., Horn 1965). It was argued that declarative knowledge tests under the consideration of various content domains can serve as a good marker for Gc, in addition to existing measurement efforts focusing on language-related abilities (Ackerman 2000; Amthauer et al. 2001). In fact, latent factors for verbal abilities and fact knowledge were found to be correlated near unity (Schipolowski et al. 2014), which supports this view. Indeed, measurement instruments used for indicating Gc increasingly employ broadly sampled tests of fact knowledge André (Beauducel and Kersting 2002; Schipolowski et al. 2014; Schroeders et al. 2020), depicting a broad factor of Gc covering knowledge that individuals accumulate during their lifetime through acculturation and learning (Cattell 1971, 1987).

Recent studies investigating the psychometric structure of Gc suggest a higher-order model, with a general factor of declarative knowledge capturing the strong positive manifold of broadly sampled content domains such as natural sciences, life sciences, humanities, and social sciences modeled as first-order factors (Steger et al. 2019). This is in line with the view that it is pivotal to conduct a broad assessment of declarative knowledge rather than administering a single in-depth and domain-specific test (Ackerman 1996, 2000). Taken together, Gc should therefore best be measured with adequate broadness with respect to its knowledge domains (Ackerman 2000; Steger et al. 2019).

### 1.2. Assessment Methods of Declarative Knowledge

Measuring declarative knowledge seems simple: administer fact knowledge questions which refer to knowledge that persons can actually acquire during their lives (Wilhelm and Schroeders 2019). The basic assumption of administering such questions is that they tap into what persons can in principle know. Apart from bearing in mind individual sample characteristics such as age (Li et al. 2004; Watrin et al. 2022), education levels (Ackerman 1996), or even characteristics of specific item samples, such as item samples suited especially for children in primary school (Schroeders et al. 2016), research on overarching psychological dispositions should preferably transcend the methods of inquiry we apply. That is, Gc should go beyond the specificities of a single test and one important way to abstract from specificities is to vary presumably irrelevant attributes of measures. It is an open question whether or not the response format of fact knowledge questions is irrelevant in the sense that they might change what a fact knowledge test measures.

Generally, two response modes for measuring Gc (and other abilities) can be distinguished: selected-response formats such as multiple-choice (MC) formats and so-called constructed-response (CR) formats (e.g., Traub 1993). Both types differ with regard to the format in which participants respond, but both depend on an item-stem that articulates a stimulus (usually a question) awaiting a response. In the case of declarative knowledge, the item-stem is usually a question with only one veridical solution. Whereas the MC format provides several response alternatives per question, of which the correct one has to be chosen (Downing and Haladyna 2006), the CR format does not provide response alternatives and usually requires test takers to retrieve the correct response from memory (e.g., Martinez 1999; Sam et al. 2018, 2019; Traub 1993). Other tests based on CR formats can require subjects to build a response by assembling given pieces of information (e.g., certain reasoning tasks Becker et al. 2015).

### 1.3. MC Format Items vs. CR Format Items

MC-format items are used for measuring a broad variety of abilities in many and diverse applied test settings (e.g., SAT since 1937, Hancock 1994; university exams, Lindner et al. 2021; PISA, Rodriguez 2003; driving license tests) since their first large scale applications through Robert Yerkes in the Army Alpha Intelligence Tests during World War I (Chan and Kennedy 2002).

Their popularity is indicative of their inherent advantages (Chan and Kennedy 2002; Walstad 2001): most saliently cost-efficient development (Abu-Zaid and Khan 2013) and administration (both paper-pencil and computerized; Schipolowski et al. 2014; Watrin et al. 2022, respectively); easily automated scoring that is not subject to reliability issues inherent in many other response formats (e.g., Kennedy and Walstad 1997); and finally, administration with simple and swift-to-deliver instructions due to the intuitive response format is easy and does not require costly training of proctors. In turn, these advantages of MC-format items can be understood as the disadvantages of CR formats (e.g., Chan and Kennedy 2002).

MC tests in general have been shown to yield good reliabilities and validity (Downing and Haladyna 2006) and are deemed “the workhorse of the testing enterprise throughout much of the world” (Downing and Haladyna 2006, p. 293). We suggest that this statement also pertains to MC tests assessing declarative knowledge (e.g., Schroeders et al. 2016; Steger et al. 2019). However, there are also reservations regarding MC-format tests and some studies set out to measure fact knowledge solely relying on CR item formats (Lynn et al. 2001; Lynn and Irwing 2002).

One concern regarding MC tests is that they might facilitate learning wrong information due to uncorrected errors, for example in college exams or other application contexts (Fazio et al. 2010). MC tests have also been argued to encourage guessing (Becker and Johnston 1999). The ability to infer the correct response from hints such as the provided response alternatives, or at least to eliminate individual distractors in the presence of incomplete expertise can also be considered an aspect of “test wiseness” or “test-taking ability” (Millman et al. 1965; Sabers 1975). If test takers are test-wise and possess relevant knowledge and if a test contains susceptible items, then the combination of these factors will result in higher scores; responses on MC-format items can be inferred without actually providing a question and these inferences seem to be driven by working memory, and prior knowledge (Hartung et al. 2017). Supposedly, strategies to take MC tests are widely established among test takers (Chittooran and Miles 2001; Kesselman-Turkel and Peterson 2004).

Questions about construct-equivalence across different response formats such as MC and CR are the subject of discussion (Rodriguez 2003). In contrast to the MC format, CR formats are often used in test settings, because they supposedly allow measuring more complex cognitive processes (such as applying actual knowledge or evaluating a concept, c.f. Bloom’s taxonomy, Krathwohl 2002) more readily than the MC format does (Martinez 1999; Rodriguez 2003). Supposedly, this view is supported by studies showing that test scores based on MC-format items only explained 50% of the variance in test scores based on essay-like CR-format items (Hickson and Reed 2011). Further studies either found no support for a single-factor solution across both response formats (Traub and Fisher 1977) or found no relationship at all between both response formats (Becker and Johnston 1999).

Opposed to these findings, several studies found MC-format tests and CR-format tests to be equally well indicative of one and the same underlying construct. For example, verbal and reading skills have been found to be best described by a single latent factor, indicating the high communality across test formats (Vernon 1962; Ward 1982). In addition, there is evidence for construct-equivalence between MC and CR formats in mathematical reasoning items (Traub and Fisher 1977). Further studies of numerical problem solving also revealed very high correlations (*r* = .90) between response formats (Harke et al. 1972; Horn 1966). Regarding knowledge questions, it was also shown that CR formats add little information beyond MC-format items (Lukhele et al. 1994; Thissen et al. 1994). This is supported by meta-analytical evidence for construct equivalence between response formats of MC and CR items, which also shows that if the same item-stem was used for investigating correlations between response formats, unity was almost approached (Rodriguez 2003). Lastly, evidence from two large-scale educational assessments (TIMSS and PIRLS; Mullis et al. 2007, 2008) shows that differences between the response formats with regard to their reliability and validity can be neglected (Schult and Sparfeldt 2018).

### 1.4. Cognitive Processes Underlying Different Response Formats

Responses to declarative knowledge questions are arguably not simply driven by crystallized information structures, but also through a variety of retrieval processes. These retrieval processes might vary across response formats. The correct answer to a knowledge question has to be percolated from existing information in long-term memory (e.g., Tulving and Watkins 1973; Unsworth 2019), that is, it has to be found amongst a manifold of other irrelevant information. For a knowledge question in MC format, one could argue that a response builds upon *recognition* of the correct response option. Actively retrieving information from long-term memory is required in CR response formats and this cognitive activity is best-labeled *recall* (e.g., Haist et al. 1992). Many authors argue, that CR response format items are needed to measure more complex cognitive processes such as a new combination of existing information in memory (e.g., Martinez 1999), and that MC-format items are not suited to assess more than pure recognition (Martinez 1999; Veloski et al. 1999).

The discontinuity hypothesis states that recognition and recall are two fundamentally different memory processes (Tulving and Watkins 1973). For example, recognition might be understood as one phase of the identification of a response, whereas recall requires two phases, namely an exhaustive search for a response and a proactive decision for it (Anderson and Bower 1972; Gillund and Shiffrin 1984). According to the discontinuity hypothesis, it is questionable whether two latent variables for declarative knowledge measured with either MC or CR format are strongly related. Presumably, both factors capture performance in different memory processes and do not reflect the same underlying ability.

Whether MC-format items and CR-format items measure the same underlying ability equally well, might depend on the ability that is to be measured, and on the actual format of the administered items (Hancock 1994). Accordingly, Tulving and Watkins (1973, p. 739) stated: “A critical problem of long standing in the psychological study of memory is concerned with the relation between recall and recognition. In what sense are they the same, and in what sense are they different?”. This question can be readily transferred to the study of competing response formats of declarative knowledge, such as MC formats and CR formats.

In an MC-format test with one correct response and three distractors, the guessing probability is 25%. If the distractors are additionally implausible, simply falsifying all implausible response alternatives might facilitate answering an item. This rejuvenates the question of whether the test takers of MC-format tests actually know the answer to a given item stem or if they simply recognize it after considering the incorrect distractors. If MC-format tests reward partial knowledge (i.e., recognition) just like real in-depth knowledge (i.e., recall), then test takers might learn to discard implausible or plainly wrong distractors instead of effortfully verifying the correct response from memory (Martinez 1999; Scouller 1998). Obviously, triggering such elimination strategies is not the measurement intention when MC format is used, and the degree to which this account for MC-format performance applies is a controversial topic (Coleman et al. 2010; Daneman and Hannon 2001; Fowler and Kroll 1978; Katz et al. 1990, 1991; Martinez 1999; Rost and Sparfeldt 2007; Sparfeldt et al. 2012).

Whereas MC response formats offer cues as to what the correct response to a knowledge question is (it has to be one of the four provided response alternatives), CR response formats usually do not present any cues regarding the correct response other than the question itself. However, the degree to which cues in open-ended response formats are presented can be manipulated experimentally. For example, instead of not restricting responses to a CR question at all, one could provide the first letter of the correct answer. This would limit the number of possible responses to the question and facilitate the executed retrieval processes (i.e., an exhaustive search of possible responses). With respect to pure mean-structure effects, for which one stable result is that MC-format items usually show higher solution probabilities (e.g., Chan and Kennedy 2002; Hohensinn and Kubinger 2011; Sam et al. 2018, 2019), the mean performance of such a cued open-ended response format should be located between conventional MC-format items and open-ended format items.

Compared to the MC format as the prototypical representative of selected-response formats, the CR format comes with more degrees of freedom regarding test construction. Whereas MC formats might hedge one pole of a fictional response format continuum, the CR format would cover the rest of the continuum in different manifestations, spanned from cued open-ended response formats that only require responding with one word to open-ended response formats that are akin to essays. Depending on the location of a response format on the continuum, the requirement of different cognitive processes for responding to an item-stem might change.

From a psychometric point of view, measuring declarative knowledge with distinct response formats (e.g., MC formats vs. open-ended formats), can plausibly deliver three alternative outcome scenarios: First, administering different response formats to measure the same underlying ability affects neither the mean structure of the items and thus the test, nor its covariance structure and thus what such tests measures in the first place. Second, the mean structure of items hinges upon the response format, but the covariance structure (i.e., the rank-order of individual test takers) is unaffected, which can indicate that the same underlying ability is tapped by the test, independent of the used response format. Third, in addition to the mean structure, the covariance structure is affected, which would indicate that different response formats measure different underlying cognitive abilities (e.g., Hickson and Reed 2011; Scully 2017).

### 1.5. The Present Studies

Studying declarative knowledge implicitly follows a seemingly longstanding tradition of using MC item formats (Ackerman 1996; Steger et al. 2019). With the present studies, we aimed at addressing the following questions: How do different response formats affect item difficulty? Do changes in the response format cause changes in the rank-order of individuals? Is the same ability measured across different response formats? We addressed these questions by investigating declarative knowledge items in three competing response formats.

To this end, we administered a broad test battery of declarative knowledge items across the following response formats: MC-format items with four response alternatives, an open-ended CR format, and an open-ended CR format with cues (e.g., the first letter of the correct response).

In study 1, we tested how empirical item difficulties of a Gc test are affected by response formats. We hypothesized that other things being equal MC items should be easiest because they supposedly rely on recognition of the correct response only rather than actually retrieving it from long-term memory. The cued open-ended response format should be more difficult, because it may require a more sophisticated retrieval process when mentally searching for the correct response to a knowledge question. Lastly, the open-ended item format should be more difficult to give a correct response to, because the room for possible responses is only restricted through the content of the knowledge question. The proposed mean effects should be apparent in both comparisons of the solution probability of single items, and aggregate scores of the response formats.

Importantly, it is unclear whether the response formats tap the same underlying cognitive ability. Arguably, different response formats require distinct cognitive processes (e.g., *recognition* in MC formats vs. *recall* in open-ended formats), and if this is the case, this should affect the covariance structure of tests, and thus the rank-order of individuals across response formats. In study 2, we tested for changes in covariance structure across response formats. To this end, we evaluated a series of competing measurement models. The research objectives and hypotheses were not preregistered.

## 2. Methods and Materials: Study 1

### 2.1. Participants and Procedure

Study 1 was conducted online (using *SociSurvey*). The study was advertised via mailing lists of the local university, via social media, and via a German online panel (*Respondi*). We conducted the study commensurate with the ethical guidelines provided by the German Society for Online Research (*DGOF*) and the EU General Data Protection Regulation (*GDPR*). The anonymity of the participants was guaranteed. Ethical approval was not required as per local legislation. In the online panel, participants were reimbursed according to the reimbursement rules of *Respondi*.

In total, *N* = 198 participants completed study 1. In order to ensure data quality, participants were excluded from data analysis, if they (a) indicated to have used unpermitted aids (e.g., using Google search during the study) (*n* = 36), (b) indicated to have participated without care (*n* = 16) or failed attention checks (*n* = 1). In addition, participants were removed if they were identified as outliers regarding defocusing events (i.e., changing tabs in their browsers >3 *SD*; *n* = 4). The final sample consisted of *N* = 142 participants. The mean age of the sample was *M* = 29.8 years (*SD* = 6.97 years, range = 18–37), and 45.8% were female. Approximately 49% of the sample indicated holding at least a high school degree.

### 2.2. Measures

*Declarative Knowledge.* In study 1, we assessed declarative knowledge using 72 items covering four broad knowledge domains and twelve subdomains, namely natural sciences (physics, biology, chemistry), social sciences (politics, law, economy), humanities (art, literature, music), and life sciences (medicine, nutrition, health) (c.f., Steger et al. 2019). All items were sampled from a large item pool (Steger et al. 2019). The items were sampled to cover the above-mentioned knowledge domains. Please note, that the item pool of Steger et al. (2019) was developed by reviewing existing knowledge test batteries (see Steger et al. 2019 for an overview). Additionally, the authors of the item pool ensured that items from various vocational profiles and education contexts were included in the original item pool (see Steger et al. 2019). The items we sampled from this pool for our studies were administered in three response formats:

1. MC-format items included a knowledge question (e.g., “What is the capital of Sweden?”) and four response alternatives with exactly one veridical response;
2. For open-ended format items, participants were only presented with the knowledge question and a text box for typing in the response;
3. The same was true for the cued open-ended format items, although in this particular response format, participants were additionally provided with a cue (the first letter of the correct response, or a restriction as to the range of the correct number).

Items were presented one at a time to the participants. All participants were instructed to guess the correct response in the MC format if they did not know the answer. Further, if they did not know an answer in the open-ended or cued format, they were instructed to indicate this by filling in “I do not know” or a question mark. There were no time restrictions and it was clearly stated in the instructions that participants should not use any aids for responding to the items.

All items were administered in all three response formats. All participants responded to all 72 items, but participants were randomly assigned to one of three item-sets, each consisting of a distinct mixture of items from different response formats so that each item in each response format was answered by approximately one-third of the sample (c.f., Table 1). No item was shown more than once to a participant.

Some items were piloted in different response formats (see Supplementary Materials, p. 1). Descriptive statistics for all items in all response formats across both studies (and our selection of the item pool of Steger et al. (2019)) are provided in the Supplementary Materials (SM Table S1).

*Para Data—Defocusing.* During testing, JavaScript tracked whether or not participants changed tabs or windows (similar to Diedenhofen and Musch 2017; Steger et al. 2020). The change in a tab is thought to capture the occurrence of so-called defocusing events and was found to be predictive of cheating in unproctored test settings (Steger et al. 2020). However, defocusing (i.e., changing a tab during an online test) should not be equated with cheating behavior per se. Although changing a tab during an online test session might be made to cheat, not all tab changes must indicate cheating. Further, cheating is also possible without changing tabs, for example through using a second digital device.. Therefore, we added this count variable as a covariate to all of our analyses in order to demonstrate that our results and main conclusions are barely affected by defocusing events such as tab changes. Nevertheless, we excluded participants with large amounts of defocusing events, because we take this as an indicator of inattentive responding (see Section 2.1 *Participants and Procedure* above).

### 2.3. Data Preparation

Prior to the main statistical analysis and after initial data cleaning, the data of the items administered in the open-ended and cued response formats were scored by human raters. All items were scored dichotomously. Items were scored as correct if participants indicated either the response which was deemed correct in the original MC item format as provided by the original item pool (Steger et al. 2019), or if participants indicated an alternative but still correct response (which was only possible in the open-ended response formats). Obvious spelling mistakes such as forgetting a letter were not counted as incorrect, whereas wrong names, wrong formulas, and answers that indicated not knowing the correct response were dismissed.

Two raters scored all responses for all persons independently. Beforehand, the raters were provided with acceptable and expected responses for each item. Cohen’s κ (Kappa; Cohen 1960) was used to determine the inter-rater reliability. After initial scoring, the average κ’s across the open-ended and the cued items were *M*_κ(open)_ = .97 (*SD*_κ(open)_ = .07) and *M*_κ(cued)_ = .97 (*SD*_κ(cued)_ = .07), respectively, indicating minor deviations of the two scorers. These minor deviations could be clarified, so that the final scoring of the open-ended data yielded in full agreement of the scorers (*M*_κ(open)_ = 1 and *M*_κ(cued)_ = 1).

### 2.4. Statistical Analysis

All analyses were conducted with R (R Core Team 2022). To make all analyses reproducible, we provide all materials necessary, including data and analysis scripts, in an online repository: https://osf.io/pse3w/.

In order to compare the difficulties of the items between response format conditions, we built means for all item-sets in all response format conditions and computed Cohen’s *d* as a measure of effect size (Cohen 1969). Additionally, in study 1 we used logistic mixed regression models. The items of each response format provide a dichotomous independent variable indicating correctness. Thus, we analyzed our data by means of logistic regression models, in which the correctness of any given item was predicted by response format (i.e., MC vs. cued open-ended vs. open-ended). In addition to the fixed effect of this predictor, we specified a full model that included the main effect of defocusing to account for participants that may have been cheating, and the interaction term of response format and defocusing to account for potential differences in tab-changing dependent on the response format, a random effect of the participants (i.e., random intercept), and a random effect of the item (i.e., random intercept), because certain facts might be better known than others. As such, we accounted for the multi-level data structure. This full model was compared with more parsimonious models (i.e., dropping single effects), to evaluate each effect. Regression weights of the main effect s can be interpreted relative to the respective reference response format.

## 3. Results: Study 1

**Figure 1:**
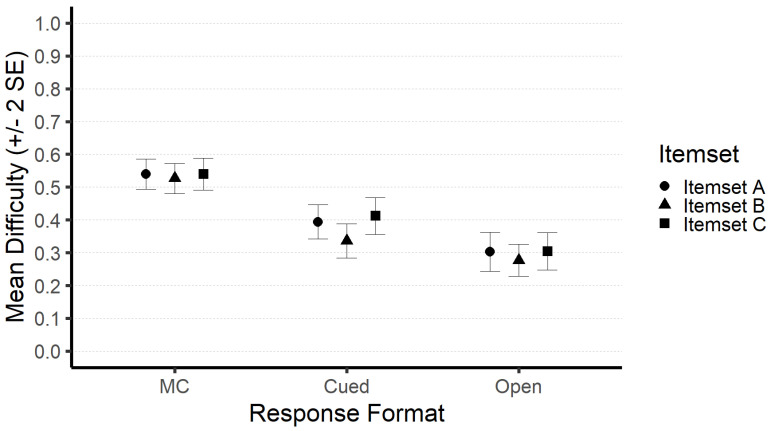
Mean difficulties of response formats for each item set.

In Figure 1, we report the mean difficulties of response formats for each itemset. In line with expectations, these statistics indicate that the MC format is easiest in each of the administered item samples. The means within one response format across item-sets are highly similar. The effect sizes for the mean differences between the response formats range between *d* = .72–1.10 for MC compared to the cued open-ended response format, *d* = 1.31–1.49 for MC compared to the open-ended response format, and *d* = .33–.57, for the cued open-ended compared to the open-ended response format, respectively. Almost all mean differences were statistically substantial (all *p* < .05; the exact test statistics can be found in the Supplementary Materials SM Table S3), with the exception of the mean difference between the cued open-ended and the open-ended response format in itemset B.

Next, we compared several logistic regression models that used the MC response format as a reference method, so that the regression weights can be interpreted relative to items of the respective reference method. We tested the full model, as described above, against two more parsimonious models that did not include (a) the random intercept for single items, and (b) the random intercept for participants. In both cases, a chi-square difference test revealed that dropping either one of the effects deteriorated model fit substantially (a: Δχ^2^(1) = 1580.9, *p* < .001, and b: Δχ^2^(1) = 799.1, *p* < .001, respectively). We thus kept both effects in the model. Additionally, we tested the full model against a model without the interaction term between response format and defocusing, an effect that implies differential effects of defocusing depending on the response format. Again, a chi-square difference test revealed that the full model fitted the data somewhat better (Δχ^2^(2) = 9.56, *p* < .01). Therefore, we also kept this interaction effect, in addition to the main effects of response format and defocusing.

Parameter estimates of the final model are presented in Table 2. The main effects are in line with expectations. Solving MC items is easier than solving cued open-ended or open-ended items. The odds in favor of solving a cued open-ended item relative to an open-ended item were less pronounced. These effects persisted if defocusing events are included in the model. The main effect of defocusing is substantial (indicating an increased probability of solving an item when defocusing events occur; c.f., Steger et al. 2020). Importantly, with and without defocusing MC format is easiest, followed by cued open-ended which in turn is easier than open-ended format. Although the fixed effect alone explained little variance in the criterion (marginal R2 = .083; Nakagawa et al. 2017), the effects of the response format were still substantial. Across the test battery of study 1, we found evidence that MC items are more frequently solved as compared with their (cued) open-ended counterparts.

An odds ratio of 1 indicates no difference between formats, whereas odds ratios > 1 indicate that the likelihood of a participant correctly responding to an item increases, relative to the reference group. Analogously, odds ratios < 1 indicate that the likelihood of a participant correctly responding to an item decreases, relative to the reference group. Please note that three items were excluded from these analyses because they were declared as outliers regarding their odds as compared to at least one of the other response formats (they exhibited ORs >3 SD from the mean distribution of ORs). Odds ratios for single items are provided in Supplementary Materials (SM Table S2).

## 4. Discussion: Study 1

We administered 72 fact knowledge items from four broad knowledge domains (Steger et al. 2019) in three response formats each. This approach allowed for a thorough test of item difficulty as a function of response format. In line with previous research, we hypothesized that different cognitive requirements might be required by more open response formats (i.e., simple *recognition* vs. more complex *recall* from long-term memory), and should be associated with lower probabilities of solving an item correctly. In other words, if surface characteristics in a diverse set of items matter, they should account for individual differences in subsets of items once the general ability is controlled for. In line with these predictions, we observed the hypothesized difficulty order of item formats. Across most items, and across all used item-sets, we found that MC-format items exhibit the highest solution probabilities, followed by the cued open-ended and concluded by the open-ended format. This rank-order might be explained by the different cognitive processes that underly solving an item of a respective response format. Whereas MC-format items naturally offer more readily accessible information through presenting test takers with alternative responses, this is not the case in the open-ended response formats—as a consequence, it is harder to solve an item in the open-ended response format and the solution probability of an item is directly associated with the a priori provided information.

It should be noted that the magnitude of all effect size estimates, on the item level, but also on the score level (that is, aggregated scores across items per participant), were relatively large, and thus the mean differences between the response formats can be deemed fairly general and stable, and cannot be easily attributed to statistical artifacts such as differences in guessing probabilities. Arithmetically, the guessing probability of the administered MC-format items was .25 per item. In contrast, the guessing probabilities of the open-ended and cued open-ended items should be approximately zero due to the inherent nature of these item formats. The arithmetic guessing of the probability of MC-format items rests on the strong assumption that all distractors (i.e., erroneous response choices) are equally attractive—however, this assumption hardly ever holds, even if item development follows the strongest quality benchmarks. As knowledge accumulation is an idiosyncratic process, the attractiveness of a distractor might also depend on person-bound characteristics, and as such, the guessing probability for MC-format items can only hardly be accounted for. Although 3-parameter IRT-models might be fit to control for arithmetic guessing probabilities by estimating a specific parameter for it, these models require large sample sizes than our Study 1 provides and are objectionable for a number of reasons, including the above-mentioned (see, e.g., Chapelle 1999).

Nevertheless, we argue that further studying guessing effects to exclude this as an explanation for the here presented results is warranted. Empirically, guessing effects could be studied across different instantiations of knowledge items that are mixed within-subject administration (i.e., between-subjects). These instantiations could span from items in an open-ended response format, over a cued open-ended format, towards individually increasing the number of response alternatives for a given question (i.e., one attractor and one distractor; one attractor and two distractors, etc.). These data could then be used to obtain an approximate estimate of what effect any given arithmetic guessing probability has on a specific item pool.

## 5. Methods and Materials: Study 2

### 5.1. Participants and Procedure

Just like Study 1, Study 2 was conducted online (using *SociSurvey*) using the same online panel (*Respondi*). We determined the sample size for Study 2 a priori (*using simsem*; Pornprasertmanit et al. 2021), based on considerations of several parameter estimates for our target latent factor models (i.e., minimally expected effect sizes and typical loadings of indicators). Across various competing models, we found that a sample size of *N* = 307 yields sufficient power (>.80, α = .001) for all targeted parameter estimates (including factor loadings and factor correlations). In total, *N* = 376 participants were included in study 2. Participants were excluded from data analysis if they (a) indicated to have used unpermitted aids (*n* = 39), (b) indicated to have participated without care (*n* = 19), or (c) failed attention checks (*n* = 1). Again, participants were removed if they changed tabs extensively during testing (>3 *SD*; *n* = 7). In addition, *n* = 2 persons encountered technical difficulties so no responses were saved in their data files. Lastly, after scoring and computing test scores, *n* = 8 participants were identified as multivariate outliers and thus removed from further analysis. The final sample size was *N* = 300. 70.3% of the sample indicated to be female; the mean age was *M* = 29.53 years (*SD* = 7.04, range = 18–60). The majority of the sample (72.3%) indicated having at least a high-school degree.

### 5.2. Measures

*Declarative Knowledge.* In Study 2, we used the same 72 items for assessing declarative knowledge as in Study 1 but administered three item sets fixed to one response format each (i.e., 24 items per response format; c.f., Table 1). There was no item overlap between the response formats. Presentation for all 72 items, and hence the response formats, was randomized for each participant in order to prevent possible confounding through fixed item sequences. The broad knowledge domains were balanced in each item set., i.e., they were represented equally across the number of items and the response formats. For each response format, items were allocated considering the recommendation that declarative knowledge scales should cover a wide range of difficulty (Schneider and McGrew 2018). We thus chose items according to both their empirical difficulties as indicated through means and standard deviations of an MC response format item pool (*N* > 1000, Steger et al. 2019), and according to their empirical difficulties within the respective response formats of Study 1.

*Para Data—Defocusing.* For Study 2, we used the same procedure to detect defocusing events as described in Study 1 (see Section 2.1 *Participants and Procedure* of Study 1 above).

### 5.3. Data Preparation

We applied the same scoring procedure for the items of Study 2 as described for Study 1. The data of both open-ended response formats in Study 2 were also scored by several human raters. In contrast to Study 1, one person rated all open-ended items, whereas another person rated all cued open-ended items. In addition to that, a third person rated all items of both response formats. The inter-rater reliability after initial scoring was very high (M_κ(open)_ = .99, SD_κ(open)_ = .01; M_κ(cued)_ = .99, SD_κ(cued)_ = .02). After clarifying deviations full agreement for all items was reached.

### 5.4. Statistical Analysis

Again, all analyses were conducted with R (R Core Team 2022) and we provide all materials necessary to reproduce the analyses in an online repository: https://osf.io/pse3w/.

In Study 2, we aimed at comparing competing measurement models to address the dimensionality of Gc across response formats. We built parcels to reduce model complexity, after establishing the unidimensionality of the single response format scales (Little et al. 2002). We thus decided to exclude items with problematic item characteristics from the item sample. We first examined item difficulties and excluded MC items below the guessing probability for the MC format (i.e., .25). In addition, we examined the corrected item-test-correlations for all response formats and excluded items with *r*_it_ < .18. After that, we computed unidimensional measurement models per subscale and excluded items with non-significant factor loadings (*lambda*), or items which considerably deteriorated model fit (*misfit*). We then built four parcels per response format maintaining domain specificity according to the broad knowledge domains (Cole et al. 2016; Steger et al. 2019). In total, *n* = 8 items were excluded from subsequent analyses (see SM Table S5 for an overview of the measurement models on the item level). In addition, please note that we provide a full correlation matrix, including descriptive statistics, for all the indicators used in our measurement models in the Supplementary Materials (SM Table S4).

*Confirmatory Factor Analysis* (CFA) was carried out by the R package *lavaan* (Rosseel 2012). Where possible, we used *full information maximum likelihood* estimation under the assumption of missing completely at random to combine missing data and parameter estimation in a single step (Schafer and Graham 2002; Enders 2010). Models based on dichotomous indicators are based on the *Weighted Least Squares Means and Variance* adjusted (WLSMV) estimator (Beauducel and Herzberg 2006); in this case, pairwise observations were used. Models based on continuous indicators are based on a maximum likelihood estimator with robust standard errors (MLR). The following fit statistics were considered to indicate good model fit: CFI (*Comparative Fit Index*) ≥ .95, RMSEA (*Root Mean Square Error of Approximation*) ≤ .06, and SRMR (*Standardized Root Mean Square Residual*) ≤ .08 (Hu and Bentler 1999). For acceptable model fit these boundaries were used: CFI ≥ .90, RMSEA ≤ .08, and SRMR ≤ .10 (Bentler 1990; Browne and Cudeck 1992). We used McDonald’s ω as an indicator of factor saturation (McDonald 1999; Raykov and Marcoulides 2011). The factor saturation of a factor indicates how much variance is accounted for by a latent variable in all underlying indicators (Brunner et al. 2012). We specified all measurement models with and without the defocusing covariate (please see the Supplementary Materials SM Figures S1–S4).

## 6. Results: Study 2

### Modeling Declarative Knowledge and Accounting for Response Formats

**Figure 2:**
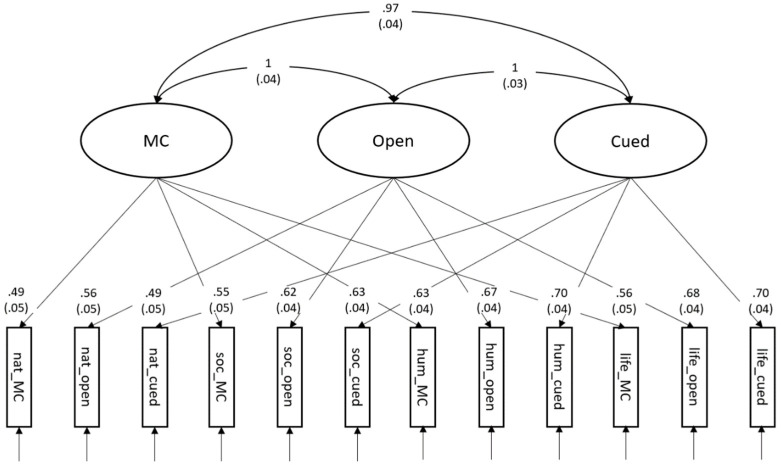
Correlated factors model of the response formats. All parameters are standardized. *n* = 300; χ^2^(51) = 148.70, CFI = .917, RMSEA = .080, SRMR = .045. Standard errors are depicted in parentheses. Please see SM Figure S1 for the model with defocusing.

We compared competing measurement models to address the dimensionality of Gc across response formats (see Table 3). The first model specified correlated factors with one latent factor per response format (Figure 2; model A in Table 3). The model fit was acceptable. The correlation between Open and Cued was estimated slightly above unity indicating extreme collinearity between the response formats. In this model, individual differences in Gc are modeled as being due to response formants exclusively. The model negates the existence of knowledge domains and allows for different rank-orders of subjects across response formats. This model shows extremely high multi-collinearity between the different response formats, which shows that the rank order of subjects does not change across response formats. This model was extended with a manifest defocusing count variable as a predictor of the correlated group factors (χ^2^(60) = 164.02, CFI = .914, RMSEA = .076, SRMR = .045; c.f., SM Figure S1) and found that defocusing was a positive predictor of all three factors (β_MC_ = .28 (*SE* = .06), β_Open_ = .36 (*SE* = .04), β_Cued_ = .31 (*SE* = .07), respectively).

Next, we specified a model in which all indicators were subsumed below a single general factor (model B in Table 3), which corresponds with the idea that response formats play no role in the covariance structure of a declarative knowledge test. A χ^2^-difference test, indicated that both models were not different (Δχ^2^(3, *N* = 300) = 2.43, *p* = .49). The general factor captured substantial variance (*p* < .001). The fit of the model was only acceptable. We extended this model with a manifest defocusing count variable as a predictor of the general factor as well (χ^2^(65) = 168.15, CFI = .914, RMSEA = .073, SRMR = .046; c.f., SM Figure S2) and found that defocusing was a positive predictor of the general factor (β = .32 (*SE* = .06)).

Next, we established a correlated factors model with four factors for the four broad knowledge domains (model C in Table 3). The four factors captured individual differences across response formats and within knowledge domains. The model negates the existence of individual differences due to response formats and also does not allow for an overarching Gc factor. Instead knowledge domains are specified as distinct but correlated entities that do not adhere to a hierarchy of cognitive abilities. Therefore, this model depicts individual differences in knowledge domains, which are independent of the administered response formats. This model fitted the data significantly better than a single general factor (Δχ^2^(6, *N* = 300) = 82.23, *p* < .001). All factors captured substantial shares of variance (ω_Nat_ = .55, ω_Soc_ = .74, ω_Hum_ = .78., and ω_Life_ = .74, respectively). Overall, the correlations between the group factors were large (all *r* > .7; *p* < .001), but did not reach unity. The model fit was good.

We added a manifest defocusing count variable to model C (χ^2^(56) = 67.04, CFI = .991, RMSEA = .026, SRMR = .028) as a predictor of all group factors (natural sciences, social sciences, humanities, life sciences) in order to control for defocusing events (c.f., SM Figure S3). The standardized regression weights were β_Nat_ = .42 (*SE* = .07), β_Soc_ = .30 (*SE* = .07), β_Hum_ = .27 (*SE* = .07), β_Life_ = .24 (*SE* = .06), respectively. Defocusing accounted for 17.4%, 9.2%, 7.5%, and 5.5% of the variance of the factors, respectively. The remaining variances of the residuals of the latent factors (i.e., individual differences after controlling for defocusing) were still significant (all *p* < .001), and correlations between factors remained unaffected. This model is included in Supplementary Materials (SM Figure S3).

Next, we established a higher-order factor model with a general factor capturing the covariance of the four latent trait factors (natural sciences, social sciences, humanities, and life sciences; see model D in Table 3 and SM Figure S4). A higher-order factor explicitly represents the overarching ability (Gc), as proposed in contemporary models of intelligence structure (c.f. Carroll 1993; McGrew 2009). This model implies that individual differences in the different knowledge domains are driven by more general differences in declarative knowledge and that these individual differences are independent of differences in the administered response format. Although this model cannot be statistically tested against model C, because these models are not nested, model fit was sufficient for both models, so preferring the higher-order factor model due to its parsimony was justified. The higher-order factor captured substantial shares of variance (*p* < .001) and the factor saturation of the higher-order factor was high (ω_Gc_ = .89; the reliability estimate was computed according to Brunner et al. 2012). Please note that we specified the same model with age as a predictor of Gc, and found that age was not a substantial predictor of Gc (β = .13, *p* = .073; *n* = 300; χ^2^(61) = 112.44, CFI = .958, RMSEA = .053, SRMR = .042).

**Figure 3:**
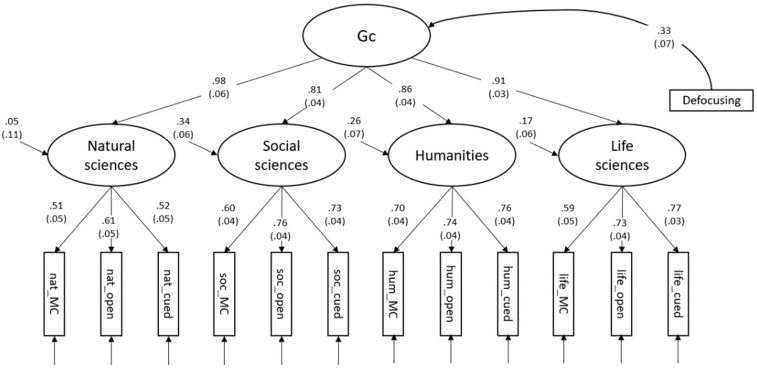
Higher-order model of the broad knowledge domains across response formats with defocusing as predictor. All parameters are standardized. *n* = 300; χ^2^(61) = 80.17, CFI = .984, RMSEA = .032, SRMR = .032. Standard errors are depicted in parentheses.

Again, we added a defocusing count variable to the model (χ^2^(61) = 80.17, CFI = .984, RMSEA = .032, SRMR = .032) as a predictor of the higher-order Gc factor. The standardized regression weight was β = .33 (*SE* = .07). Defocusing, therefore, accounted for 11% of the variance in the higher-order factor for crystallized intelligence. The remaining variance (i.e., individual differences after controlling for defocusing) was still significant (*p* < .001). The model is provided in Figure 3.

Eventually, we decided to further elaborate the higher-order factor model (model D of Table 3) by allowing for method variance due to the use of indicators of different response formats. We computed an additional model where we added two method factors to the model supposed to capture the joint variance of the open-ended and the cued open-ended response format. Theoretically, the nested method factors should explain individual differences in the indicators that are not already explained by the (domain-specific) knowledge factors. We chose the MC format as the reference method because this method is widely used across a range of applied fields. In sum, the model still fitted the data well (*n* = 300; χ^2^(41) = 45.32, CFI = .996, RMSEA = .019, SRMR = .025). The method factors were not able to account for any substantial shares of variance in the indicators and showed poor factor saturation (ω_Cued_ = .27; ω_Open_ = .34). We compared this model (method factors included) to the higher-order model (model D in Table 3) by a χ^2^-difference test, which indicated that both models were not significantly different (Δχ^2^(9, *N* = 300) = 16.65, *p* = .055), and thus favoring the more parsimonious model without the proposed method factors. In sum, this means that it is sufficient to explain individual differences in the indicators with latent factors that are independent of the administered response format.

## 7. Discussion: Study 2

In study 2, we took advantage of the fact, that the 72 knowledge items from study 1 were homogenous regarding mean differences across response formats. A common supposition is that CR format items measure some cognitive processes such as remembering specific information from memory (i.e., knowledge) more readily than MC items (Martinez 1999; Rodriguez 2003). In fact, it was argued that CR format items even tap creative processes, because some items require new combinations of existing information (Schult and Sparfeldt 2018). If this were the case, the covariance between CR and MC tests should be reliably below unity. In study 2, we checked for the effects of manipulating response formats on the covariance structure of declarative fact knowledge items by means of confirmatory latent variable modeling. As predicted, we confirmed and replicated that the mean structure is substantially affected by the response format. However, the rank-order of participants was unaffected by altering the response format. In other words, correlations between latent factors for MC and CR response formats reached unity. Individual differences in the knowledge tests were due to knowledge domains, which in turn could be subsumed below an overarching Gc factor. Response format turned out to be irrelevant to account for individual differences. From a multi-trait multi-method perspective (Campbell and Fiske 1959), method factors turned out to be irrelevant, whereas trait factors accounted for all of the individual differences.

## 8. General Discussion

We administered a broad fact knowledge test in two studies of the general population in three response formats: an MC format, a cued open-ended format, and an open-ended format. We tested how the empirical difficulties of the administered items were affected by response formats, and whether the rank-order of individuals taking the test changed across divergent response formats. Taken together, our results suggest that different response formats affect the mean structure of items, but the rank-orders of individuals remain the same.

### 8.1. Response Formats as Means to an End

Although the MC format is widely used (Chan and Kennedy 2002), it has been challenged in the literature many times (e.g., Becker and Johnston 1999; Hickson and Reed 2011; Krieg and Uyar 2001), and recommendations for using CR items as the superior item format are prevalent (e.g., Hickson and Reed 2011; Martinez 1999; Veloski et al. 1999).

From a measurement perspective, both the MC and the CR response formats should be understood as tools of measurement and therefore as specific means to an end. The question of whether both tools achieve their purpose equally well, in our case the assessment of declarative knowledge, has not yet been conclusively answered.

We identified three competing positions, from which different patterns of results can be derived: First, neither the mean structure nor the covariance structure of a test is affected by using different response formats. Second, only the mean-structure is affected by the response formats. Third, both the mean- and the covariance structure are affected by the response formats.

From an individual differences perspective, tests differing in their response formats would perform equally well, if the rank-order of individuals remains the same (Ackerman and Hambrick 2020). If the rank-order of individuals would change, this would indicate that the tests measure something different. If common assumptions that different response formats tap different forms of cognition (e.g., Buckles and Siegfried 2006) were true, abilities and methods for measuring them would be confounded.

Given the results, we conclude that measuring declarative knowledge with different response formats is possible without any loss of information regarding the rank-order of individuals. Although the mean structure was clearly affected by using different response formats, the rank-order of individuals did not change as a function of how we measured declarative knowledge. Although we cannot rule out that distinct cognitive processes are involved in answering fact knowledge questions in different response formats, as is predicted by the discontinuity hypothesis (Tulving and Watkins 1973), our data show that this is a concern that can be neglected if one is interested in individual differences. Clearly, correctly responding to fact knowledge items is a question of prior knowledge, but the possible underlying cognitive processes of identifying or retrieving a veridical solution to a knowledge question was irrelevant in accounting for individual differences in fact knowledge, which is in line with other recent studies on the subject (e.g., Schult and Sparfeldt 2018).

As all three administered response formats were indistinguishable psychometrically, some pragmatic considerations might lead to favoring one format over the other. From an individual differences perspective, the costs and benefits of response format are mostly a methodological concern. For example, response formats could be seen as something that affects how easy tests can be instructed and scored, which relates to their being economically sound. In some instances, different response formats might be deemed different in terms of some reliability concepts. For example, we could ask how reliable a 20 items MC test is relative to an equally lengthy CR format test, but we could also ask how reliable a 20 min test session with MC items is relative to an equally long session with CR items. In terms of scoring, MC-based tests have a clear advantage. On the other side, battling with how guessing probability should be figured in has plagued generations of psychometricians.

### 8.2. Recognition, Recall, or What to Study Next

From a cognitive perspective, the distinction between recognition and recall is the most salient and striking feature that presumably distinguishes MC and CR item types (and in our case, the three administered response formats). Different response formats would then come along with different cognitive requirements. Often, these requirements are studied by comparing means in recognition and recall sessions with comparable materials, either within or between subjects. However, materials typically used in experiments that juxtapose recognition and recall are stimulus-sets over which experimental control can be exerted. For instance, intuitively it makes sense to ensure that stimulus-sets are completely new for all participants so that they can all go through a controlled learning phase prior to showing recognition or recall. This novelty can, for example, be ensured by creating artificial or meaningless stimulus-sets. This is not the case for a test battery of declarative knowledge items, because these items have to be meaningful by definition. Whether or not recognition and recall as measured by virtue of a declarative fact knowledge test with different response formats is psychometrically relevant (i.e., for explaining individual differences), is an empirical question. The present data suggest that response formats are not nearly as important as often suggested. A study allowing for latent variable modeling of declarative fact knowledge and broad retrieval abilities both measured with recognition and recall methods could show whether or not the results we report here extend to settings often used in experimental psychology.

Thus, broad retrieval abilities (Gr; Schneider and McGrew 2018) should be considered in further studies evaluating possible differences between MC format and CR format items. Broad retrieval abilities are understood as the rate and fluency with which individuals can selectively retrieve and produce information stored in long term-memory (Schneider and McGrew 2018). Established determinants of general retrieval ability are declarative knowledge (c.f., Hakstian and Cattell 1978; Jewsbury and Bowden 2017; Unsworth 2019) and working memory capacity (e.g., Rosen and Engle 1997; Unsworth 2019; Unsworth et al. 2011). As both prototypical retrieval tasks and open-ended declarative knowledge items require free production (i.e., responses to questions), a multivariate study investigating broad retrieval abilities, and declarative knowledge in MC and the here-used CR response formats could inform us further about whether different cognitive processes are tapped with different item formats for measuring declarative knowledge. Based on the present results, we predict that broad retrieval abilities are equally important for both types of item formats.

In addition to that, prior work shows that broad retrieval abilities can be explained through individual differences in working memory capacity (e.g., Rosen and Engle 1997). In turn, fluid intelligence is also strongly predicted by working memory capacity (Oberauer et al. 2005), due to the load put on working memory in prototypical reasoning tasks. The mean structures from the current studies suggest that different cognitive processes in responding to items might be tapped. Whereas the MC format urges test takers to recognize the correct response by comparing the provided response alternatives to what the test takers might have stored in memory, the open-ended formats lead them to exhaustively and proactively search for the correct response. Arguably, the latter process puts more demand on working memory, as the individuals have to bear in mind the responses they have already discarded. This makes sense and is in line with previous literature showing that WMC is needed for controlled searches in long-term memory (e.g., Unsworth and Brewer 2009). In contrast, the MC format already provides some particular response alternatives, and these do not have to be kept in mind, because they are accessible to the respondents without any cognitive effort. Therefore, in contrast to CR formats, MC formats have been argued to have the smallest risk of being confounded by fluid intelligence (Schipolowski et al. 2014), because they impose lower demands on working memory.

Please note that the irrelevance of the response format for explaining the covariance structure, as observed in the present study, has an important implication. If performance in MC response format items, which supposedly tap recognition, and open-ended response format tests, which arguably tap recall, are perfectly correlated, they cannot be differentially related with other variables. For instance, recall tests might be argued to stress working memory more strongly by virtue of requiring subjects to maintain a list of candidate responses they discard as erroneous. However, the present results are at odds with such differential relations. Therefore, it might be argued that the here-presented results deviate from previous findings stressing that recognition and recall can be psychometrically distinguished (e.g., Unsworth and Brewer 2009; Unsworth 2019). However, it is important to note that differences in operationalizing vague terms such as “recognition” and “recall” should be considered when interpreting the present data. Studies in which recall (also termed familiarity) and recognition (also termed recollection) can be psychometrically distinguished (e.g., Unsworth and Brewer 2009) rely on tasks measuring memory for previously learned lists (e.g., word lists). The present study presupposes an understanding of the terms “recognition” and “recall” in the context of fact knowledge, which intertwines recognition and recall with the general knowledge that is either existent in long-term memory or not. Caution should thus be exerted in directly comparing results based on different study paradigms.

In fact, the term “recognition” suggests that the solution to MC items is stored in a verbatim fashion in LTM and that the distractors do not compete in being recognized. Both assumptions are likely to be wrong. It is implausible that question-response pairs are deposited in LTM waiting to be triggered by a recognition question one day. Often enough responses to fact knowledge questions require elaborate questions and also lengthy responses. The term recognition, therefore, downplays what is performed in such MC questions. In some instances, it might be recognition of a response that is stored in a verbatim fashion in LTM. In other instances, solving MC items requires weighting response options in terms of plausibility or probability prior to responding. In still other instances, the correct solution might be inferred by eliminating distractors. In yet other instances, a lucky punch by pure guessing might be what is observed. Needless to say, these and many different paths to a response are unlikely to be fixed for an item. Whereas some persons might simply retrieve a solution, others might be guessing, and still others might weigh the evidence of each response option and the roles of these participants might change in the next MC question.

This might also be an effect of item content. Future research should investigate whether different items might be more or less associated with semantic or episodic memory and if this predicts how difficult they are to solve when accounting for individual learning histories. Fact knowledge questions should refer to knowledge that persons can actually acquire during their lives (Wilhelm and Schroeders 20219, but as this is an idiosyncratic aspect, differences between response formats with respect to which kind of information the items tap might be considered. Such a study could be conducted via an experience-sampling method with several groups, where, across time, the different groups are exposed to different learning environments tapping different topics. After an a priori determined time of several weeks with relatively constant learning histories, the newly acquired knowledge of the participants could be tested. To then determine whether the individual learning history of the participants plays a role in responding to certain items from certain knowledge domains in competing response formats, all participants will be not only tested with regard to their, in light of the study, personal learning history, but also on all topics from the other experimental groups.

In addition to that, future research could test whether the correlation between open-ended and MC item formats might hinge upon the age of the test takers. If the association between these response formats changed as a function of age, this would indicate that the cognitive processes underlying the response behavior might be subject to change, too; which might correspond to a decline in retrieval ability in advanced age.

### 8.3. Limitations

The present studies feature important strengths such as the administration of a broad test battery of declarative knowledge to two independent samples of the general population, and the use of suitable modeling approaches (e.g., logistic mixed regression and latent variable models). A key limitation of the present research designs is that both studies were conducted online and unproctored. These limitations should introduce noise into measurement which should make it more difficult to find reliable associations. However, the key result we report is a perfect correlation of latent variables for recognition (i.e., MC items) and recall (i.e., open-ended and cued open-ended items). We made effort to remove invalid observations, for example by using a control mechanism to observe whether participants changed tabs during the test sessions (Diedenhofen and Musch 2017). Nevertheless, future research should replicate the present results in a proctored laboratory test setting in an effort to contribute to replicable and cumulative research (Flake and Fried 2020). Lastly, it should be noted that our results, especially regarding the construct validity, do not necessarily transfer to measurements of other cognitive abilities for which different response formats can be administered (e.g., reasoning ability as measured with matrix construction tests; c.f., Becker et al. 2015, 2016).

## 9. Conclusions

The effects of response formats on tests of declarative fact knowledge showed expected mean differences but no distinction on the level of latent variables. If they have the option to choose, researchers and practitioners should use MC-format items, as this response format is easier to administer and cheaper to score, and declarative fact knowledge does not change as a function of the response format. Rather, individual differences in declarative fact knowledge are best expressed as a high-order factor model with broad domain factors below an overarching Gc factor.

## Figures and Tables

**Table 1:** Design of the studies.

Study	Group (*N*)	*n* (Items) + Itemset			Total Items
		MC	Cued Open-Ended	Open-Ended	
1	1 (*N* = 46)	24 A	24 C	24 B	72
	2 (*N* = 50)	24 B	24 A	24 C	72
	3 (*N* = 46)	24 C	24 B	24 A	72
2	1 (*N* = 300)	24	24	24	72

*Note*. The reported sample sizes are after data exclusion. Each item set of Study 1 (a, b, c) contained different items and the item sets were allocated to different response formats across the groups of Study 1.

**Table 2:** Logistic regression model with standardized regression weights and odds ratios (OR).

Model		With Defocusing		
MC Reference	Fixed Effects	Estimate (*SE*)	*OR*	*p*
	(Intercept)	.22 (.15)	1.25	.15
	Cued	−.98 (.06)	.38	<.001
	Open	−1.55 (.07)	.21	<.001
	Defocusing	.65 (.18)	1.91	<.001
	Defocusing*Cued	.24 (.21)	1.28	.24
	Defocusing*Open	.61 (.21)	1.85	<.01
	Random effect	Variance (*SD*)		
	(Intercept of Person)	.76 (.87)		
	(Intercept of Item)	1.20 (1.10)		

**Table 3:** Competing measurement models.

Measurement Model		χ^2^	df	CFI	RMSEA	[90% CI]	SRMR
A	3 Correlated Factors Response Formats	148.7	51	.917	.080	[.065; .095]	.045
B	g-factor	150.90	54	.918	.077	[.063; .092]	.045
C	4 Correlated Factors Knowledge Domains	57.87	48	.992	.026	[.000; .048]	.026
D	Higher-Order Knowledge Domains	62.98	50	.989	.029	[.000; .050]	.029

*Note.* All models are based on *N* = 300 participants and on *n* = 12 indicators.

## Data Availability

To make all analyses reproducible, we provide all materials necessary, including data and analysis scripts, in an online repository: https://osf.io/pse3w/.

## Supplementary Materials

The following supporting information can be downloaded at: https://www.mdpi.com/article/10.3390/jintelligence10040102/s1.
