# Supplementary material for: Stop Worrying about Multiple-Choice: Fact Knowledge Does Not Change with Response Format
Source: J Intell. 2022 Nov 14;10(4):102. doi: 10.3390/jintelligence10040102 (PMC9680349; doi:10.3390/jintelligence10040102)
Supplement: Supplementary file 1 [file jintelligence-10-00102-s001.zip › jintelligence-1867776-supplementary.pdf]

Stop Worrying About Multiple-Choice: Fact Knowledge Does Not Change With Response Format

Supplementary Material

Method

Pilot Study

Please note that some of the items were used in a pilot study, where we sought to obtain some first difficulty estimates for an open-ended and a cued response format for the single items. In this pilot study, we tested N = 161 participants (n = 33 students and n = 128 *Prolific* members; M_{age} = 32.78). In the pilot study, we administered 60 items of the item pool from Steger and colleagues (2019) covering the same broad knowledge domains (natural sciences, social sciences, life sciences, and humanities). However, not all participants worked on all items, or on all response formats, respectively. Prior to responding to an item, participants were presented only with the knowledge question and were asked to indicate whether they knew the answer, whether they did not know the answer, or whether they would know the answer if they were presented with a cue. Participants stating that they would know the answer to a question were referred to the item in the open-ended response format, whereas participants stating they might know the answer if presented with a cue were referred to the cued open-ended item format. Participants stating not to know an answer were referred to an MC response format and instructed to guess. The number of participants working on a specific item in the cued open-ended item format ranged from n = 9-36 and in the open-ended format from n = 3-126. After the pilot study, we replaced some of the items, which were either too easy (i.e., all participants solved it correctly) or too difficult (i.e., no participants solved it correctly). Additionally, we decided to add more items to our scale for the main study to obtain a broader assessment of the construct.

Results

SM Table S1

Descriptive Statistics of All Gc Items.

	MC					Cued				Open			
	Steger et al. (2019)	Study 1 (*N*=46-50)		Study 2 (*N* = 300)		Study 1 (*N*=46-51)		Study 2 (*N* = 300)		Study 1 (*N*=45-50)		Study 2 (*N* = 300)	
Item	*M*	*M*	*SD*	*M*	*SD*	*M*	*SD*	*M*	*SD*	*M*	*SD*	*M*	*SD*
bio013	0.72	0.48	0.50			0.13	0.34			0.04	0.21	0.05	0.23
bio036	0.33	0.26	0.44	0.39	0.49	0.16	0.37			0.22	0.42		
bio067	0.47	0.33	0.47			0.87	0.34	0.92	0.27	0.56	0.50		
bio068	0.92	0.76	0.43			0.72	0.46			0.59	0.50	0.67	0.47
bio083	0.56	0.52	0.50			0.33	0.47			0.20	0.40	0.16	0.37
bio084	0.87	0.78	0.42	0.75	0.43	0.64	0.48			0.43	0.50		
bio088	0.78	0.65	0.48			0.20	0.40	0.22	0.42	0.12	0.33		
che014	0.36	0.44	0.50			0.09	0.28			0.02	0.15	0.02	0.15
che023	0.48	0.35	0.48	0.50	0.50	0.22	0.42			0.11	0.31		
che031	0.99	0.85	0.36			0.61	0.49	0.60	0.49	0.56	0.50		
che035	0.95	0.80	0.40	0.88	0.33	0.26	0.44			0.24	0.43		
che050	0.41	0.54	0.50	0.57	0.50	0.34	0.48			0.26	0.44		
che072	0.45	0.36	0.48			0.63	0.49			0.22	0.42	0.21	0.41
che101	0.49	0.46	0.50			0.24	0.43	0.26	0.44	0.24	0.43		
nut053	0.3	0.33	0.47			0.17	0.38	0.11	0.32	0.12	0.33		
nut074	0.99	1.00	0.00	0.99	0.10	0.92	0.27			0.67	0.47		
nut081	0.45	0.37	0.49	0.39	0.49	0.16	0.37			0.07	0.25		
nut089	0.48	0.46	0.50			0.30	0.47			0.26	0.44	0.34	0.47
hea008	0.57	0.50	0.51			0.35	0.48			0.39	0.49	0.31	0.46
hea056	0.99	0.96	0.20			0.54	0.50			0.65	0.48	0.69	0.46
hea057	0.6	0.41	0.50			0.33	0.47	0.35	0.48	0.20	0.40		
hea063	0.79	0.61	0.49			0.48	0.51	0.54	0.50	0.42	0.50		
hea064	0.97	0.96	0.21	0.95	0.21	0.86	0.35			0.87	0.34		
hea091	0.94	0.94	0.24			0.78	0.42			0.70	0.47	0.79	0.41
law012	0.84	0.35	0.48			0.22	0.42	0.08	0.27	0.20	0.40		
law015	0.56	0.26	0.44			0.39	0.49	0.34	0.47	0.12	0.33		
law029	0.92	0.89	0.31	0.91	0.29	0.68	0.47			0.50	0.51		
law068	0.84	0.74	0.44			0.43	0.50			0.37	0.49	0.32	0.47
law081	0.36	0.26	0.44	0.38	0.49	0.02	0.14			0.13	0.34		
law105	0.35	0.50	0.51			0.61	0.49	0.71	0.46	0.18	0.39		
art007	0.43	0.26	0.44			0.15	0.36	0.20	0.40	0.16	0.37		
art008	0.35	0.13	0.34	0.31	0.46	0.22	0.42			0.22	0.42		
art017	0.53	0.57	0.50	0.54	0.50	0.12	0.33			0.17	0.38		
art018	0.57	0.39	0.49			0.09	0.28	0.17	0.37	0.18	0.39		
art041	0.92	0.65	0.48			0.36	0.48	0.57	0.50	0.22	0.42		
art047	0.87	0.91	0.28			0.63	0.49			0.56	0.50	0.25	0.43
art028	0.88	0.52	0.50	0.65	0.48	0.24	0.43			0.20	0.40		
lit024	0.59	0.46	0.50	0.49	0.50	0.28	0.45			0.22	0.42		

lit040	0.52	0.44	0.50			0.41	0.50			0.49	0.51	0.48	0.50
lit042	0.88	0.57	0.50			0.33	0.47	0.44	0.50	0.20	0.40		
lit046	0.93	0.70	0.47	0.83	0.37	0.30	0.46			0.46	0.50		
lit051	0.95	0.62	0.49			0.67	0.47			0.17	0.38	0.22	0.42
lit124	0.36	0.34	0.48			0.46	0.50			0.07	0.25	0.15	0.36
med002	0.3	0.32	0.47			0.17	0.38			0.13	0.34	0.22	0.42
med009	0.54	0.43	0.50	0.45	0.50	0.42	0.50			0.41	0.50		
med047	0.85	0.72	0.46	0.77	0.42	0.28	0.45			0.48	0.51		
med060	0.44	0.20	0.40			0.15	0.36	0.23	0.42	0.14	0.35		
med062	0.9	0.87	0.34			0.61	0.49	0.73	0.44	0.46	0.50		
med078	0.63	0.57	0.50			0.09	0.28	0.02	0.15	0.02	0.14		
med093	0.57	0.39	0.49	0.49	0.50	0.74	0.44			0.35	0.48		
med096	0.65	0.46	0.50			0.57	0.50			0.39	0.49	0.33	0.47
mus015	0.45	0.22	0.42	0.30	0.46	0.04	0.20			0.00	0.00		
mus021	0.39	0.28	0.45			0.52	0.51			0.28	0.46	0.18	0.38
mus040	0.45	0.43	0.50			0.72	0.46	0.68	0.47	0.32	0.47		
mus043	0.62	0.38	0.49			0.72	0.46			0.43	0.50	0.44	0.50
mus046	0.84	0.83	0.38			0.11	0.31	0.27	0.44	0.12	0.33		
phy057	0.44	0.41	0.50			0.28	0.46	0.38	0.49	0.30	0.46		
phy075	0.81	0.56	0.50			0.24	0.43			0.35	0.48	0.26	0.44
phy090	0.56	0.43	0.50	0.46	0.50	0.08	0.27			0.22	0.42		
phy109	0.53	0.78	0.42			0.89	0.31	0.90	0.30	0.42	0.50		
pol029	0.46	0.48	0.50			0.33	0.47			0.28	0.46	0.26	0.44
pol034	0.73	0.63	0.49			0.59	0.50	0.64	0.48	0.35	0.48		
pol048	0.79	0.72	0.45			0.63	0.49			0.54	0.50	0.57	0.50
pol058	0.65	0.41	0.50	0.52	0.50	0.30	0.46			0.20	0.40		
pol070	0.32	0.28	0.46	0.24	0.43	0.10	0.31			0.07	0.25		
pol104	0.49	0.38	0.49			0.13	0.34			0.09	0.28	0.08	0.28
eco029	0.53	0.30	0.46			0.00	0.00			0.02	0.15	0.04	0.20
eco038	0.72	0.63	0.49			0.02	0.15	0.02	0.14	0.00	0.00		
eco050	0.89	0.83	0.38	0.87	0.34	0.46	0.50			0.50	0.51		
eco062	0.84	0.68	0.47			0.50	0.51			0.39	0.49	0.49	0.50
eco086	0.88	0.74	0.44			0.70	0.47	0.80	0.40	0.66	0.48		
eco090	0.48	0.52	0.51	0.54	0.50	0.10	0.30			0.30	0.47		

Note. bio = biology, che = chemistry, nut = nutrition, hea = health, law = law, art = art, lit = literature, med = medicine, mus = music, phy = physics, pol = politics, eco = economics. Biology, chemistry, and physics belong to the broad knowledge domain of natural sciences. Economics and politics belong to the broad knowledge domain of social sciences. Literature, art, and music belong to the broad knowledge domain of humanities. Nutrition, medicine, and health belong to the broad knowledge domain of life sciences.

SM Table S2

Odds Ratios for All Items (Study 1).

Item	MC vs. Cued			Cued vs. Open			MC vs. Open		
	OR	95%-CI		OR	95%-CI		OR	95%-CI	
		lower	upper		lower	upper		lower	upper
bio013	0.17	0.06	0.45	0.32	0.04	1.54	0.05	0.01	0.2
bio036	0.55	0.19	1.49	1.45	0.51	4.24	0.79	0.29	2.09
bio067*	13.08	4.78	41.4	0.2	0.06	0.53	2.59	1.14	6.11
bio068	0.8	0.32	2.03	0.56	0.23	1.35	0.45	0.18	1.09
bio083	0.45	0.19	1.03	0.51	0.19	1.32	0.23	0.09	0.56
bio084	0.5	0.19	1.23	0.44	0.19	0.99	0.22	0.08	0.54
bio088	0.13	0.05	0.34	0.57	0.17	1.75	0.08	0.02	0.21
che014	0.13	0.03	0.38	0.26	0.01	1.97	0.03	0	0.17
che023	0.53	0.21	1.32	0.44	0.13	1.36	0.24	0.07	0.69
che031	0.29	0.1	0.76	0.82	0.36	1.86	0.23	0.08	0.61
che035	0.09	0.03	0.23	0.9	0.35	2.29	0.08	0.03	0.21
che050	0.44	0.19	1	0.69	0.28	1.67	0.3	0.12	0.72
che072	2.98	1.31	7.03	0.17	0.06	0.41	0.5	0.19	1.23
che101	0.38	0.15	0.92	1	0.39	2.62	0.38	0.15	0.91
ern053	0.44	0.16	1.17	0.78	0.25	2.41	0.34	0.12	0.93
ern074	NA	NA	NA	0.19	0.05	0.58	NA	NA	NA
ern081	0.33	0.12	0.86	0.38	0.07	1.45	0.13	0.03	0.42
ern089	0.52	0.22	1.2	0.81	0.32	2.03	0.42	0.17	0.99
gsu008	0.54	0.23	1.22	1.2	0.51	2.85	0.65	0.28	1.46
gsu056	0.05	0.01	0.21	1.56	0.67	3.69	0.08	0.01	0.33
gsu057	0.69	0.29	1.63	0.52	0.2	1.32	0.36	0.14	0.89
gsu063	0.59	0.26	1.36	0.79	0.35	1.79	0.47	0.2	1.06
gsu064	0.3	0.04	1.35	1.08	0.32	3.71	0.32	0.04	1.54
gsu091	0.24	0.05	0.87	0.64	0.24	1.65	0.15	0.03	0.53
jur012	0.53	0.2	1.33	0.9	0.33	2.47	0.47	0.18	1.19
jur015	1.8	0.74	4.49	0.26	0.09	0.68	0.47	0.16	1.31
jur029	0.27	0.08	0.77	0.48	0.2	1.09	0.13	0.04	0.36

jur068	0.28	0.11	0.64	0.77	0.33	1.77	0.21	0.09	0.5
jur081*	0.07	0	0.37	6.5	1.01	173.35	0.43	0.14	1.26
jur105	1.55	0.67	3.59	0.15	0.05	0.36	0.23	0.08	0.56
kun007	0.52	0.17	1.45	1.22	0.41	3.77	0.63	0.23	1.68
kun008	1.85	0.63	5.95	0.99	0.36	2.64	1.82	0.6	5.96
kun017	0.11	0.04	0.29	1.53	0.48	5.14	0.17	0.06	0.42
kun018	0.16	0.04	0.47	2.55	0.77	10.25	0.4	0.15	0.97
kun028	0.31	0.13	0.7	0.5	0.19	1.23	0.15	0.06	0.38
kun041	0.17	0.04	0.52	0.75	0.33	1.71	0.13	0.03	0.38
kun047	0.3	0.12	0.7	0.78	0.28	2.13	0.23	0.09	0.56
lit024	0.47	0.2	1.09	0.72	0.27	1.84	0.34	0.13	0.83
lit040	0.9	0.39	2.03	1.41	0.62	3.27	1.27	0.56	2.87
lit042	0.38	0.16	0.88	0.52	0.2	1.32	0.2	0.08	0.48
lit046	0.19	0.08	0.45	1.94	0.84	4.59	0.37	0.15	0.87
lit051	1.26	0.54	2.97	0.11	0.04	0.27	0.13	0.05	0.34
lit124	1.62	0.71	3.75	0.12	0.03	0.36	0.19	0.05	0.59
med002	0.45	0.16	1.18	0.72	0.21	2.3	0.33	0.11	0.9
med009	0.94	0.42	2.14	0.97	0.43	2.21	0.92	0.4	2.11
med047	0.16	0.06	0.38	2.33	1	5.56	0.37	0.15	0.86
med060	0.74	0.24	2.23	1.06	0.34	3.35	0.79	0.26	2.3
med062	0.24	0.08	0.66	0.55	0.24	1.24	0.13	0.04	0.35
med078	0.08	0.02	0.23	0.24	0.01	1.81	0.02	0	0.1
med093	4.33	1.85	10.65	0.19	0.08	0.45	0.83	0.35	1.96
med096	1.52	0.68	3.45	0.5	0.21	1.15	0.76	0.33	1.71
mus015	NA	NA	NA	NA	NA	NA	NA	NA	NA
mus021	2.76	1.19	6.62	0.37	0.15	0.86	1.01	0.41	2.5
mus040	3.24	1.37	7.96	0.19	0.08	0.45	0.62	0.26	1.42
mus043	4.05	1.74	9.9	0.31	0.13	0.73	1.25	0.55	2.86
mus046	0.03	0.01	0.09	1.11	0.3	4.26	0.03	0.01	0.09
phy057	0.56	0.23	1.35	1.09	0.45	2.67	0.61	0.26	1.43
phy075	0.25	0.1	0.6	1.68	0.68	4.3	0.42	0.18	0.96
phy090	0.12	0.03	0.36	3.09	0.93	12.46	0.37	0.14	0.9

phy109	2.23	0.71	7.93	0.09	0.03	0.26	0.21	0.08	0.5
pol029	0.53	0.23	1.21	0.82	0.33	2.01	0.43	0.18	1
pol034	0.84	0.36	1.95	0.4	0.17	0.91	0.34	0.14	0.76
pol048	0.67	0.28	1.59	0.7	0.3	1.62	0.47	0.2	1.09
pol058	0.61	0.26	1.43	0.57	0.21	1.47	0.35	0.13	0.89
pol070	0.35	0.11	1.01	0.53	0.1	2.2	0.19	0.04	0.65
pol104	0.25	0.08	0.68	0.65	0.15	2.5	0.16	0.04	0.49
wir029	NA	NA	NA	NA	NA	NA	NA	NA	NA
wir038	0.02	0	0.08	0.92	0.02	36.57	0.01	0	0.07
wir050	0.18	0.07	0.46	1.17	0.52	2.64	0.22	0.08	0.55
wir062	0.48	0.2	1.09	0.65	0.28	1.48	0.31	0.13	0.71
wir086	0.81	0.32	2.03	0.85	0.35	2.02	0.69	0.28	1.67
wir090*	0.11	0.03	0.3	3.82	1.3	13.11	0.41	0.17	0.95

Note. Items marked with a * were excluded from analyses, because scoring the responses to these items were ambiguous. If values are NA, then the computation of odds ratios was not possible due to extreme responses in one of the two corresponding item formats (i.e., either all participants solved an item, or no participant solved an item). bio = biology, che = chemistry, nut = nutrition, hea = health, law = law, art = art, lit = literature, med = medicine, mus = music, phy = physics, pol = politics, eco = economics. Biology, chemistry, and physics belong to the broad knowledge domain of natural sciences. Economics and politics belong to the broad knowledge domain of social sciences. Literature, art, and music belong to the broad knowledge domain of humanities. Nutrition, medicine, and health belong to the broad knowledge domain of life sciences.

SM Table S3

Mean differences between test scores per response format of the single itemsets in Study 1.

	Response Formats		*Mean (SD)*	*Mean (SD)*					
Itemset	X	Y	X	Y	*t*	*df*	*p*	*d*	*d* 95%-CI
	MC	Cued	.54 (.16)	.39 (.18)	4.2	88.7	< .001	.87	[.43; 1.30]
A	MC	Open	.54 (.16)	.30 (.20)	6.3	84.95	< .001	1.3	[.85; 1.77]
	Cued	Open	.39 (.18)	.30 (.20)	2.3	88.6	< .05	.48	[.06; .90]
	MC	Cued	.53 (.16)	.34 (.19)	5.4	96.35	< .001	1.09	[.66; 1.51]
B	MC	Open	.53 (.16)	.28 (.17)	7.4	97.68	< .001	1.48	[1.03; 1.93]
	Cued	Open	.34 (.19)	.28 (.17)	1.6	97.46	.10	.33	[-.07; .72]
	MC	Cued	.54 (.16)	.41 (.19)	3.5	88.13	< .001	.72	[.29; 1.14]
C	MC	Open	.54 (.16)	.30 (.19)	6.3	87.76	< .001	1.32	[.86; 1.78]
	Cued	Open	.41 (.19)	.30 (.19)	2.7	89.98	< .01	.57	[.14; .99]

Note. d = Cohen’s d. All provided descriptive statistics in this table refer to Figure 1 of the manuscript.

SM Table S4

Means, standard deviations, skew, kurtosis, and bivariate correlations for all indicators used in the measurement models of Study 2.

	Variable	*M*	*SD*	Skew	Kurtosis	1	2	3	4	5	6	7	8	9	10	11	12
1	gc.mc_nat	.59	.24	-.17	-.58	1											
2	gc.mc_soc	.71	.24	-.51	-.31	.27***	1										
3	gc.mc_hum	.52	.24	.23	-.66	.35***	.36***	1									
4	gc.mc_life	.53	.26	.00	-.65	.28***	.28***	.31***	1								
5	gc.open_nat	.23	.18	.72	.17	.30***	.32***	.29***	.37***	1							
6	gc.open_soc	.34	.26	.41	-.79	.30***	.46***	.34***	.31***	.37***	1						
7	gc.open_hum	.31	.25	.48	-.66	.31***	.38***	.53***	.35***	.37***	.45***	1					
8	gc.open_life	.40	.28	.27	-.69	.31***	.33***	.41***	.43***	.40***	.40***	.42***	1				
9	gc.cued_nat	.67	.21	-.06	-.70	.29***	.27***	.26***	.23***	.31***	.26***	.35***	.37***	1			
10	gc.cued_soc	.43	.21	-.12	-.70	.31***	.42***	.32***	.30***	.31***	.56***	.40***	.39***	.29***	1		
11	gc.cued_hum	.39	.25	.39	-.38	.30***	.31***	.56***	.38***	.33***	.39***	.53***	.49***	.31***	.47***	1	
12	gc.cued_life	.33	.23	.29	-.84	.34***	.27***	.39***	.46***	.48***	.41***	.42***	.56***	.37***	.43***	.49***	1
13	Defocusing	.07	.12	2.31	5.03	.20***	.14*	.17**	.13*	.27***	.27***	.27***	.13*	.20***	.21***	.17**	.23***

Note. *** $p < .001$; ** $p < .01$; * $p < .05$

SM Table S5

Measurement Models of the Response Formats.

Measurement Model		*n* (Indicators)	*n* (Persons)	χ^2	df	CFI	RMSEA	[90% CI]	SRMR	ω	exclusion due to
1a	MC Items	23	308	382.94	230	.659	.047	[.038; .055]	.121	.65	guessing (1 item)
1b	MC Items	19	308	182.6	152	.920	.026	[.004; .038]	.093	.67	misfit (2 items), low r_{it} (2 items)
2a	Open Items	24	308	336.97	252	.907	.033	[.023; .042]	.118	.78	
2b	Open Items	22	308	261.97	209	.940	.029	[.016; .039]	.109	.79	misfit (1 item), low r_{it} (1 item)
3a	Cued Items	24	308	352.03	252	.915	.036	[.027; .045]	.132	.81	
3b	Cued Items	23	308	305.45	230	.932	.033	[.022; .042]	.129	.80	misfit (1 item)

Note. ω = McDonald’s Omega (McDonald, 1999). Please note that one item for the MC scale had to be excluded from the scale initially, because the item was below guessing probability and had an $r_{it} < .05$. The unidimensional measurement model did not converge with the item included. We excluded MC items below the corresponding guessing probability for the MC format (i.e., *guessing*; cutoff-value for considering an item answered with guessing probability .25). In addition to that, we examined the corrected item-test-correlations for all response formats and excluded items with $r_{it} < .18$ from the scales (low r_{it}). After that, we computed unidimensional measurement models per subscale and excluded items which considerably deteriorated model fit (*misfit*).

SM Figure S1

Correlated Factors of the Response Formats with Defocusing.

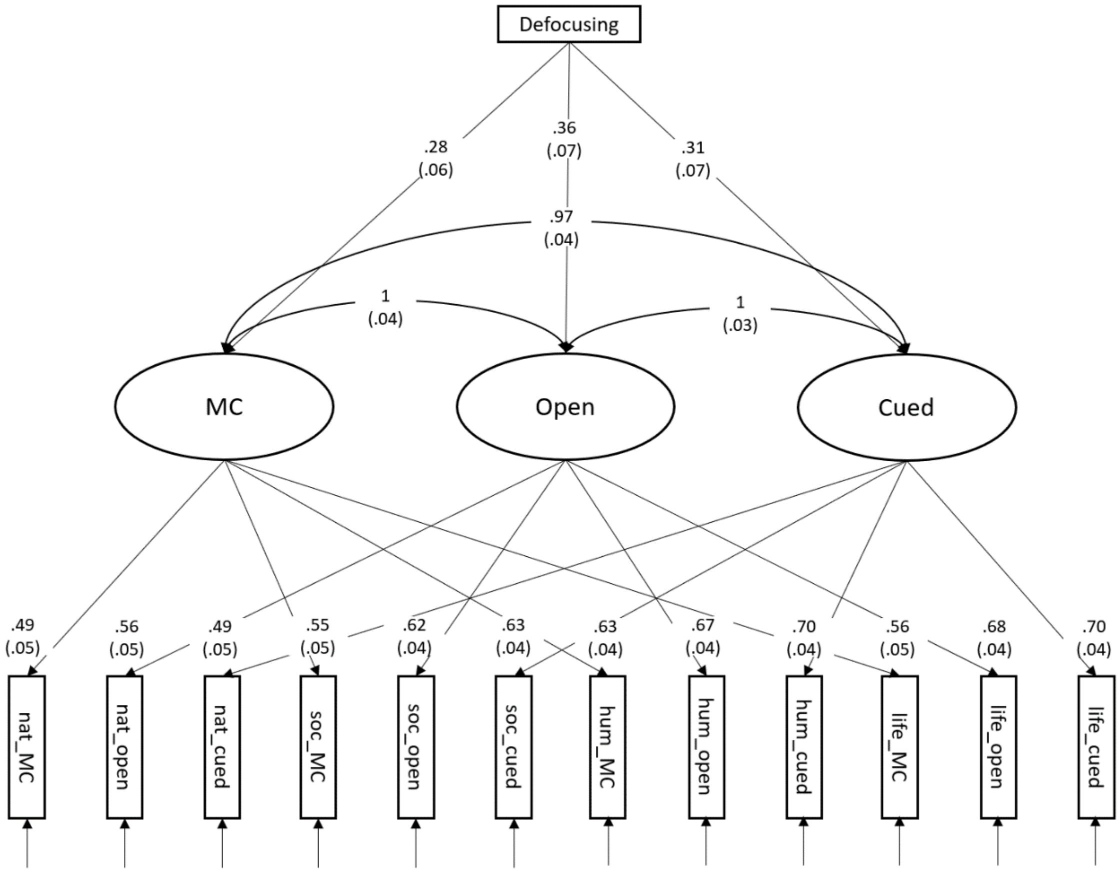

Note. All parameters are standardized. *n* = 300; $\chi^2(60) = 164.02$, CFI = .914, RMSEA = .076, SRMR = .045. Standard errors are depicted in parentheses. Please note that this model is an extension of model A of Table 3 in the manuscript.

SM Figure S2

A General Factor Model with Defocusing.

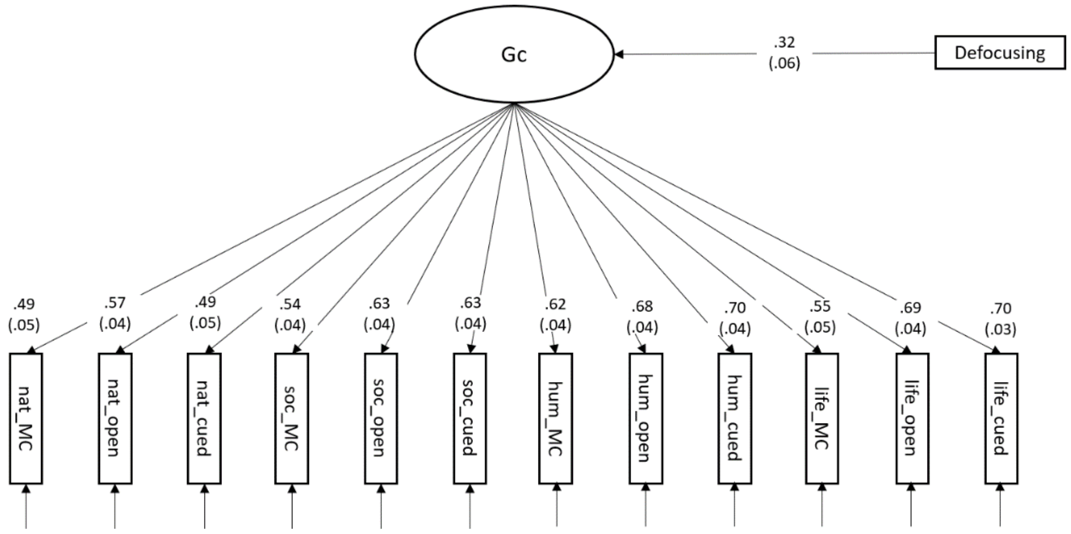

Note. All parameters are standardized. n = 300; $\chi^2(65)$ = 168.15, CFI = .914, RMSEA = .073, SRMR = .046. Standard errors are depicted in parentheses. Please note that this model is an extension of model B of Table 3 in the manuscript.

SM Figure S3

Correlated Factors of the Broad Knowledge Domains Across Response Formats.

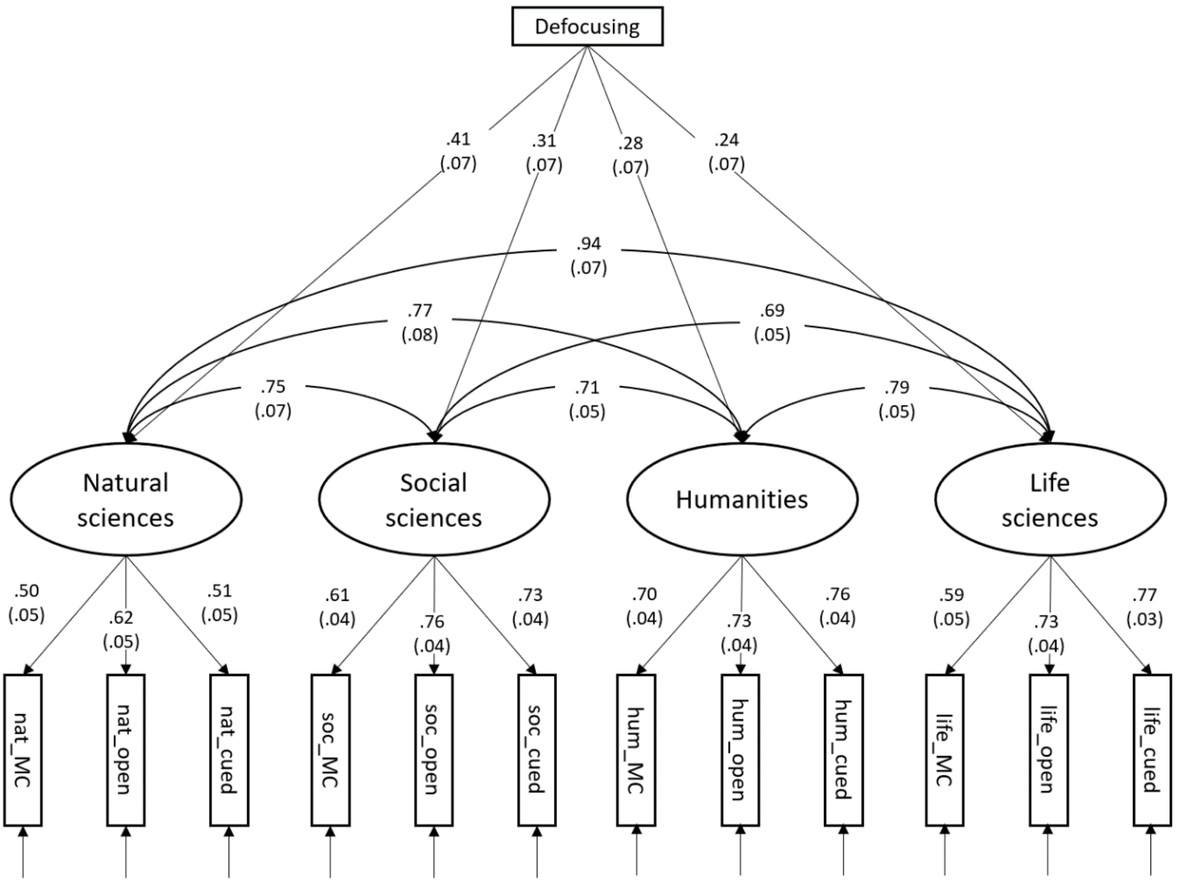

Note. All parameters are standardized. n = 300; $\chi^2(56)$ = 67.04, CFI = .991, RMSEA = .026, SRMR = .028. Standard errors are depicted in parentheses. Please note that this model is an extension of model C of Table 3 in the manuscript.

SM Figure S4

Higher-Order Model of the Broad Knowledge Domains Across Response Formats.

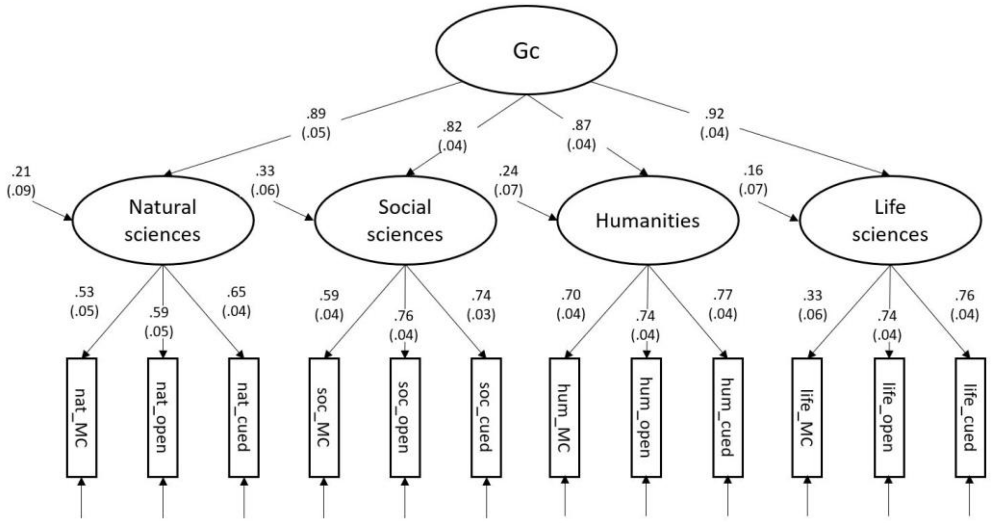

Note. All parameters are standardized. n = 300; $\chi 2(50) = 62.98$, CFI = .989, RMSEA = .029, SRMR = .029. Standard errors are depicted in parentheses. Please note that this model a depiction of model D of Table 3 in the manuscript. Please note that we specified the same model with age as a predictor of Gc, and found that age was not a substantial predictor of Gc ($\beta = .13$, $p = .073$; $n = 300$; $\chi 2(61) = 112.44$, CFI = .958, RMSEA = .053, SRMR = .042).

References

McDonald, R. P. (1999). *Test theory: A unified treatment.* Erlbaum.

Steger, D., Schroeders, U., & Wilhelm, O. (2019). On the dimensionality of crystallized intelligence: A smartphone-based assessment. *Intelligence*, *72*, 76–85.
